# The effects of training with loads that maximise power output and individualised repetitions vs. traditional power training

**DOI:** 10.1371/journal.pone.0186601

**Published:** 2017-10-20

**Authors:** J. M. Sarabia, M. Moya-Ramón, J. L. Hernández-Davó, J. Fernandez-Fernandez, R. Sabido

**Affiliations:** Sports Research Centre, Universidad Miguel Hernández de Elche, Elche, Spain; University of Rome, ITALY

## Abstract

**Background:**

It has been suggested that strength training effects (i.e. neural or structural) vary, depending on the total repetitions performed and velocity loss in each training set.

**Purpose:**

The aim of this study is to compare the effects of two training programmes (i.e. one with loads that maximise power output and individualised repetitions, and the other following traditional power training).

**Methods:**

Twenty-five males were divided into three groups (optimum power [OP = 10], traditional training [TT = 9] and control group [CG = 6]). The training load used for OP was individualised using loads that maximised power output (41.7% ± 5.8 of one repetition maximum [1RM]) and repetitions at maximum power (4 to 9 repetitions, or ‘reps’). Volume (sets x repetitions) was the same for both experimental groups, while intensity for TT was that needed to perform only 50% of the maximum number of possible repetitions (i.e. 61.1%–66.6% of 1RM). The training programme ran over 11 weeks (2 sessions per week; 4–5 sets per session; 3-minute rests between sets), with pre-, intermediate and post-tests which included: anthropometry, 1RM, peak power output (PPO) with 30%, 40% and 50% of 1RM in the bench press throw, and salivary testosterone (ST) and cortisol (SC) concentrations. Rate of perceived exertion (RPE) and power output were recorded in all sessions.

**Results:**

Following the intermediate test, PPO was increased in the OP group for each load (10.9%–13.2%). Following the post-test, both experimental groups had increased 1RM (11.8%–13.8%) and PPO for each load (14.1%–19.6%). Significant decreases in PPO were found for the TT group during all sets (4.9%–15.4%), along with significantly higher RPE (37%).

**Conclusion:**

OP appears to be a more efficient method of training, with less neuromuscular fatigue and lower RPE.

## Introduction

Strength and power are considered critical components of modern athletic performance [1] and health [2]. More specifically, power output is an important attribute in determining athletic ability and predicting success in different sports [1], as well as improve mobility-related outcomes in older adults [2]. Muscular power has been shown to be improved following either force- (e.g. heavy loads) or velocity-oriented (e.g. plyometrics) training programmes [3, 4]. Nevertheless, considerable debate exists concerning not only the most efficient method for improving power output, but also the optimal load required to optimise such power adaptations [5]. Historically, the training methods regarded as being the best for developing explosive muscular power have differed, ranging from high-resistance (i.e. >70% of one repetition maximum [1RM]), low-velocity training (strength-oriented) [6, 7]; through low-resistance (i.e. <30% of 1RM), high-velocity training (speed-oriented) [3, 8]; to intermediate-resistance (i.e. 50–70% of 1RM), high-velocity training [9, 10]. Additionally, the individualised load that elicits the highest mechanical power, referred to as the ‘optimal load’, has been suggested to be appropriate for seeking power output adaptations [5, 10]. Thus, several previous studies have suggested the use of ballistic exercises with individual loads that maximise power output as the most recommended training strategy to achieve power improvements [10, 11].

The underlying mechanisms leading to superior adaptations after training with a specific load are not clearly defined, although it is theorised that training with loads that maximise power output provides an effective stimulus for eliciting specific adaptations in the rate of neural activation [3, 8]. This can be understood from research showing both neural and muscle fibre adaptations (i.e. increasing the number of type II fibres) after training with loads that maximise power output [12–14]. In addition, previous research supporting these findings has suggested that training with maximum power output (Pmax) results in superior improvements in maximal power production compared with other loading conditions [5, 15].

However, strength training prescription involves not only intensity (% 1RM) but also the combination of several other factors, including: type of exercises used; volume (sets × repetitions); exercise sequence within a strength training session; repetition velocity; training frequency; and rest interval length between sets [5, 16]. It has been suggested that the main adaptations (i.e. neural, hypertrophic, metabolic) after a strength training programme depend on, among other factors, the total number of repetitions performed [17] and velocity loss in each training set [18]. In this regard, previous research has argued that traditional strength training leads to repetition failure, and consequently the speed of repetitions slows naturally as fatigue increases [18]. Thus, some authors have recommended that no more than 50% of the maximal number of possible repetitions against any load (e.g. six repetitions of a 12 RM load) [19–21] should be performed when training for power output development. However, this approach seems very general, and one might speculate that training in accordance with such recommendations would lead to reductions in power output production above during a set. This could deflect the training effect towards endurance, promoting undesirable effects (i.e. the stimulation of slow fibres) and failing to reach maximum power [22]. On the other hand, maintaining an optimum power approach suggests that, within each set, only the number of repetitions producing a power output above 90% of the maximum power should be executed [23].

In addition to mechanical aspects (i.e. power output), hormonal responses to strength training may play a role in the development of strength [16, 24]. However, this is still unknown. Changes in resting concentrations of hormones such as cortisol and testosterone appear to reflect the current state of muscle tissue, and changes (e.g. elevations or reductions) may occur at various stages depending on the manipulation of training parameters (i.e. volume/intensity). In power training, secretion patterns of cortisol and testosterone appear inconsistent [16]. Moreover, most previous research has used non-equivalent volumes and/or intensity training [25–27], rendering comparison infeasible.

Thus, the aim of this study was to compare the adaptations in both mechanical and hormonal variables after an eight-week strength training intervention following two different methodologies (i.e. one based on maintaining maximum mechanical power (non-power loss) with loads that maximise power output; the other following non-failure power training recommendations).

## Materials and methods

### Subjects

A total of 25 recreationally active, young, male college students (aged 19–25 years) volunteered to participate in the study. Their characteristics are summarised in Table 1. Before any participation, the experimental procedures and potential risks were fully explained to them, and written informed consent obtained (S1 File). The procedure was approved by the institutional review committee of the Miguel Hernández University (Elche, Spain) and conformed to the recommendations of the Declaration of Helsinki. Participants completed a health questionnaire, which showed all subjects to be reportedly free of any acute or chronic illness, disease or injury that would contraindicate the performance of exercise. Of the 30 originally recruited subjects, five did not complete the study and were therefore excluded from the data analysis because their training adherence was less than 81%.

**Table 1:** Descriptive data. Data are expressed by mean ± SD.

Group	N	Age (yr)	Body Mass (kg)	Height (m)	Fat Mass (%)	Lean Muscle Mass (%)
**Total**	25	21.7 ± 1.7	71.5 ± 7.7	174.7 ± 5.8	12.6 ± 4.8	44.1 ± 4.0
**OP**	9	20.8 ± 1.7	71.7 ± 7.4	172.5 ± 6.2	11.8 ± 2.8	44.2 ± 3.6
**TT**	10	22.2 ± 1.6	74.2 ± 8.0	177.5 ± 5.6	14.5 ± 7.0	42.0 ± 4.5
**CG**	6	21.9 ± 1.5	68.5 ± 7.3	173.8 ± 5.1	11.4 ± 3.3	46.2 ± 3.1

Abbreviations: OP = optimum power group; TT = traditional training group; CG = control group.

### Design

**Fig 1:**
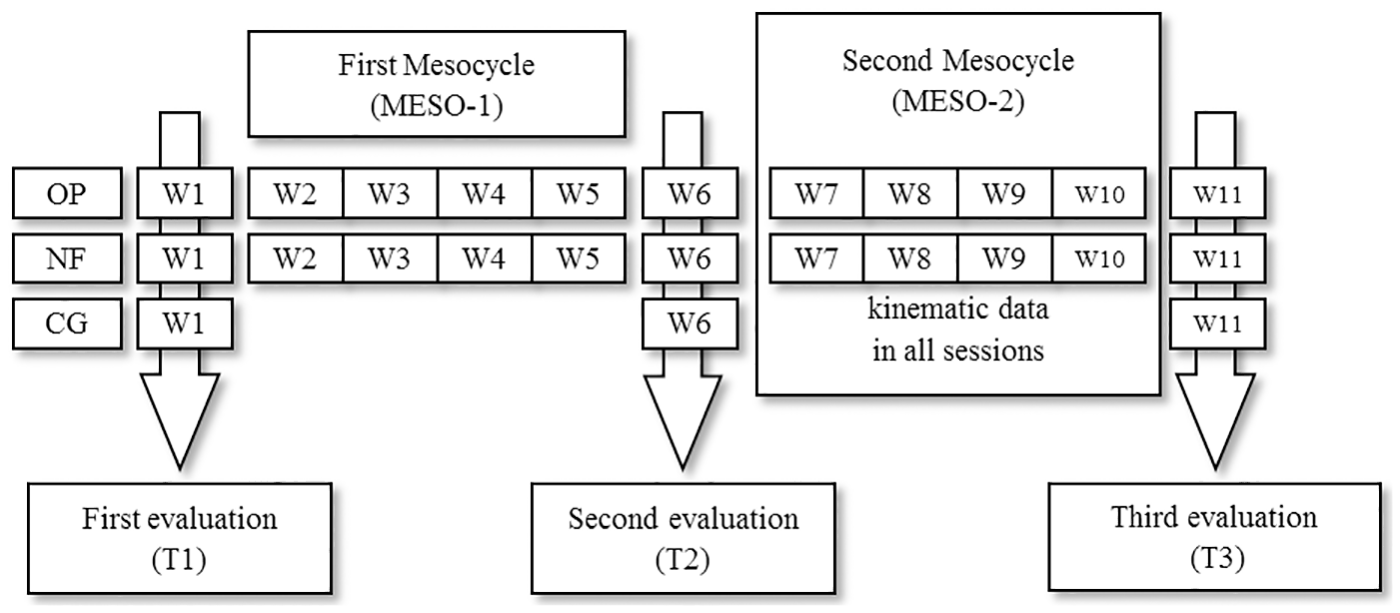
Experimental design.

A controlled and longitudinal design (i.e. pre-test and post-test) was used. Before any baseline testing, all subjects attended two familiarisation sessions to introduce the testing and training procedures and ensure that any learning effect was minimised. Pre- (T1), intermediate (T2) and post-tests (T3) included: anthropometry, 1RM, maximum concentric mechanical power with 30%, 40% and 50% of 1RM (P30, P40 and P50, respectively) in the bench press throw exercise, and one set to failure with optimal load. Salivary testosterone (ST) and cortisol (SC) concentrations were obtained during the testing weeks. Subjects were divided into three homogenous groups according to their initial 1RM values: an optimum power group (OP = 10), a traditional training group (TT = 9) and a control group (CG = 6). The training intervention consisted of 11 weeks (see Fig 1), divided into an eight-week main training programme (MTP) (divided into two mesocycles: MESO-1 and MESO-2) and three testing weeks (T1, T2 and T3). The MTP consisted of 16 sessions (2 session × week) with 48 hours of rest between sessions.

### Methodology

#### Anthropometry

The body mass and height of participants when wearing only shorts were measured to the nearest 0.1 kg and 0.1 cm respectively, using calibrated Oregon Scientific (GR101) scales and Seca Alpha stadiometer, again respectively. Skinfold, girth and breadth were determined by an accredited researcher using calibrated skinfold callipers (Holtain LTD, Crymych, UK) and following the guidelines proposed by the International Society for the Advancement of Kinanthropometry (ISAK).

#### Bench press throw tests

The 1RM test for the bench press was performed using a Smith Machine ((Multipower M953, Technogym, Gambettola, Italy). Kinematic data were recorded by attaching a rotary encoder to one end of the bar (T-Force system, Ergotech, Spain), while relevant parameters were automatically calculated using specialised software (T-Force Dynamic Measurement System) [28]. The validity and reliability of this system have been established previously [29], with intra-class correlation coefficient (ICC) values ranging from 0.93 to 0.98 [30]. The ICC values for the data in this study were > 0.90 for all variables and times. For power variables analysis, only the propulsive concentric phase was analysed. The 1RM bench press was assessed using a previously established protocol [31], which requires that participants progressively increase resistance until the 1RM is achieved. Rest period between trials was at least five minutes [32].

In the second testing session, participants performed three repetitions in their 30%, 40% and 50% of 1RM, using the bench press throw exercise in order to measure peak power output (PPO) for each load and thereby establish the Pmax [33]. Subsequently, a set to failure was performed using the optimal load for each participant, with Pmax used to determine the number of optimal repetitions for each participant.

#### Salivary cortisol and testosterone

Three saliva samples were collected on Sundays during T1, T2 and T3, at 8am, 11am and 6pm. Participants provided 5–10ml of saliva in a plastic tube with cotton (Salivette^®^, Sarstedt, France). They were instructed to collect the sample before eating or drinking. They were also told to rinse thoroughly with tap water but not to brush their teeth before taking the saliva sample, in order to avoid contamination of the saliva with blood from possible microinjuries in the oral cavity [34]. Samples were then collected and frozen at -20°C in the laboratory’s refrigerator, until assay. SC concentration was determined by enzyme-linked immunosorbent assay (ELISA), with a lower limit of sensitivity of 0.0537 μg/dl, and average intra- and inter-assay coefficients of variations of 2.61% and 7.47%, respectively.

#### Rate of perceived exertion (RPE)

RPE values were obtained immediately after the last set of each session using the OMNI–RES scale [35]. The OMNI–RES scale is a scale of exercise intensity that ranges from ‘extremely easy’ (0) to ‘extremely hard’ (10). Subjects were instructed in how to use the OMNI-RES scale, as per Robertson et al. [35].

#### Training programme

Participants performed a specific warm-up that included joint mobilisation and supine bench press with a Smith machine. Intensity for OP was individualised for each participant using loads that maximised power output in the bench press throw exercise (i.e. 41.7% ± 5.8 of 1RM in the MESO-1). Volume for OP was individualised according to the maximum number of repetitions in which the participant was able to develop more than 90% Pmax [3]. Hence, the maximum number of repetitions performed was different for each participant (ranging from 4 to 9), the average for the groups being 6.1 ± 2.6 repetitions in the MESO-1 and 5.4 ± 1.3 in the MESO-2. Volume in TT was the average of performed repetitions per set for OP (six repetitions in the MESO-1 and five in the MESO-2), in order to equalise volume in both groups. Intensity for TT was established according to the recommendation of using only 50% of possible repetitions [21]. Thus, given that all participants in TT performed the same number of repetitions per set (i.e. six for MESO-1 and five for MESO-2), the load used was that which enabled participants to perform double the prescribed repetition per sets (i.e. 12RM for MESO-1 and 10RM for MESO-2) [21]. The relative load related to these 12RM and 10RM was estimated in accordance with Legaz-Arrese et al. [23]. In both groups, the training load (intensity and volume) was adjusted in the MESO-2 based on data collected in T2 (see Table 2). During both mesocycles, both groups performed four sets in the first two weeks and five sets in the last two weeks. Recovery time between sets was three minutes for both groups [31]. Both groups performed sessions separately. Participants were verbally instructed and encouraged to perform each repetition as fast as possible without receiving performance feedback.

**Table 2:** Mean ± SD volume (repetitions) and intensity (% of 1RM) during the main training period for each experimental group.

Group	MESO-1			MESO-2		
	reps	% 1RM	Diff. OL (%1RM)	reps	% 1RM	Diff. OL (%1RM)
**OP**	6.1 ± 2.6	43.3 ± 5.0	---	5.4 ± 1.3	42.2 ± 6.7	---
**TT**	6.0	61.1 (12RM)	18.1 ± 6.7	5.0	66.6 (10RM)	21.6 ± 5.3

Abbreviations: OP = optimum power group; TT = traditional training group; MESO-1 = first mesocycle of four weeks; MESO-2 = second mesocycle of four weeks; Diff. OL = difference between loads used and loads that maximise power output.

### Statistical analyses

Standard statistical methods were used to calculate means ± SD. A one-way ANOVA was used to determine any differences among the three groups’ initial strength, power and anthropometric profile. The training-related effects were assessed by a MANOVA with repeated measures (time × groups). To analyse kinematic variables in MESO-2 sessions, data were grouped for analysis with regard to the number of sets per session and a repeated measures ANOVA applied. Where a significant difference was found for either main effect (time or group), Scheffè’s post-hoc analysis was performed to locate the pairwise differences between the means. SPSS V.22 was used for statistical calculations. Statistical significance was accepted where *p* < 0.05. Cohen’s *d* and the standardised mean difference [36] was used to calculate effect size (ES), represented by ‘*d*’ and interpreted for a recreationally trained sample according to Rhea [37] as < 0.35 (trivial), 0.35–0.80 (small), 0.80–1.50 (moderate) and >1.5 (large). The minimum difference (MD) needed to be considered real change attributable to the training was calculated for each mechanical variable, as proposed by Weir [38].

## Results

At the beginning of the training programme, no significant differences were observed between the groups on any measured variable. In addition, no significant changes in anthropometric data were found at any time, for any group.

**Fig 2:**
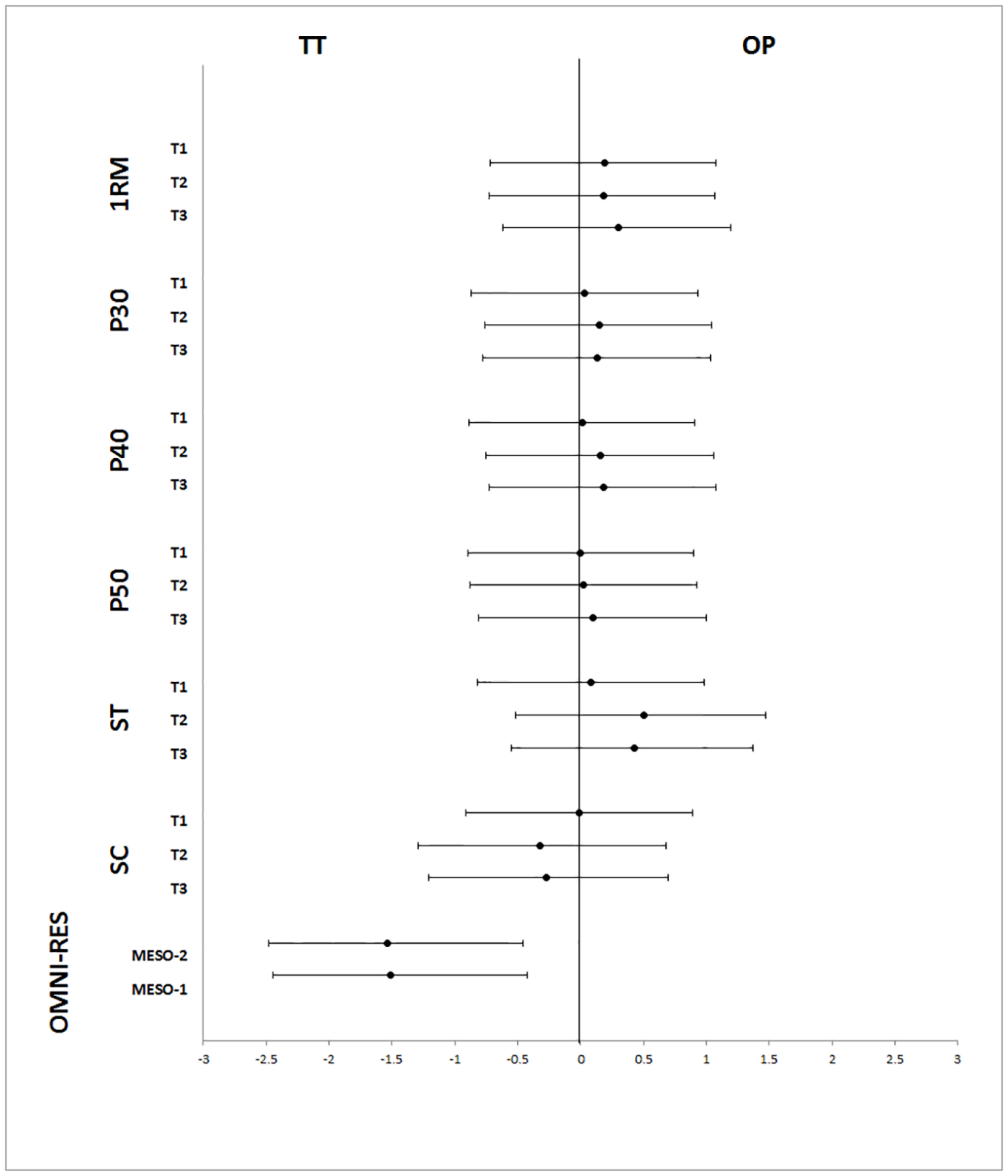
Forest plots of effect sizes for all variables at three points of assessment. Results are expressed as Cohen’s *d* with 95% confidence intervals (CIs). OP = optimum power group; TT = traditional training group; T1 = pre-intervention evaluation; T2 = evaluation after first four weeks’ training; T3 = post-intervention evaluation; 1RM = one repetition maximum; P30 = peak power output with 30% of 1RM; P40 = peak power output with 40% of 1RM; P50 = peak power output with 50% of 1RM; ST = salivary testosterone; SC = salivary cortisol; MESO-1 = mean value of OMNI-RES scale punctuation for first four weeks’ training; MESO-2 = mean value of OMNI-RES scale punctuation for second four weeks’ training.

Performance measures (1RM, P30, P40 and P50) obtained during T1, T2 and T3 are presented in Table 3. In addition, the ES and confidence intervals are shown in Fig 2. The raw data can also be viewed in S1 Table.

**Table 3:** Mean ± SD values of the performance tests during T1, T2 and T3.

		T1	T2	T3
**1RM (kg)**	**OP**	77.4 ± 19.6	81.7 ± 19.7	88.1 ± 20.0††**
	**TT**	73.9 ± 17.8	78.5 ± 16.3	82.6 ± 16.9†
	**CG**	74.9 ± 9.7	75.3 ± 9.9	76.7 ± 12.7
**P30 (watts)**	**OP**	466.7 ± 148.0	523.3 ± 148.0†*	558.1 ± 115.5††**
	**TT**	461.3 ± 168.0	502.0 ± 166.1	538.9 ± 169.5††**
	**CG**	464.8 ± 97.3	469.0 ± 85.4	469.5 ± 101.8
**P40 (watts)**	**OP**	501.8 ± 144.2	567.8 ± 138.6††*	597.9 ± 139.9††**
	**TT**	499.6 ± 164.0	543.6 ± 160.7	569.9 ± 162.1††*
	**CG**	502.0 ± 94.8	509.2 ± 89.7	520.2 ± 116.8
**P50 (watts)**	**OP**	502.6 ± 134.4	557.6 ± 164.3†	587.6 ± 149.4††
	**TT**	494.6 ± 161.8	554.0 ± 184.0†	571.4 ± 173.4††
	**CG**	511.2 ± 100.4	516.7 ± 96.9	534.2 ± 101.6

Abbreviations: OP = optimum power group; TT = traditional training group; CG = control group; T1 = pre-intervention evaluation; T2 = evaluation after first four weeks’ training; T3 = post-intervention evaluation; 1RM = one repetition maximum; P30 = peak power output with 30% of 1RM; P40 = peak power output with 40% of 1RM; P50 = peak power output with 50% of 1RM;

^†^ = significant differences from T1 p < .05;

^††^ = significant differences from T1 p < .01;

* = significant differences from CG p < .05;

** = significant differences from CG p < .01.

After MESO-1, OP showed significant improvements in P30 (*p* = .026, *d* = .38), P40 (*p* = .003, *d* = .46) and P50 (*p* = .015, *d* = .42), while TT showed significant improvements in P50 (*p* < .016, *d* = .36). Significant differences were found between OP and CG in P30 (*p* = .049, *d* = .64) and P40 (*p* = .014, *d* = .65).

After the eight-week training period, OP and TT showed significant increases in 1RM (*p* = .008, *d* = .55 and *p* = .028, *d* = .49, respectively), P30 (*p* < .000, *d* = .62, and *p* = .001, *d* = .46, respectively), P40 (*p* < .000, *d* = .67 and *p* = .001, *d* = .43, respectively) and P50 (*p* < .000, *d* = .63 and *p* = .001, *d* = .47, respectively). Significant differences were found between OP and CG in 1RM (*p* = .009, *d* = .90), P30 (*p* = .001, *d* = .87) and P40 (*p* = .001, *d* = .67). In addition, significant differences were found between TT and CG in P30 (*p* = .004, *d* = .68) and P40 (*p* = .024, *d* = .43). Nevertheless, the MD values were 9.1 kg for 1RM and 77.7 W, 76.2 W and 74.4 W for P30, P40 and P50, respectively. After the full training period, only OP showed a change higher than these MD values.

**Fig 3:**
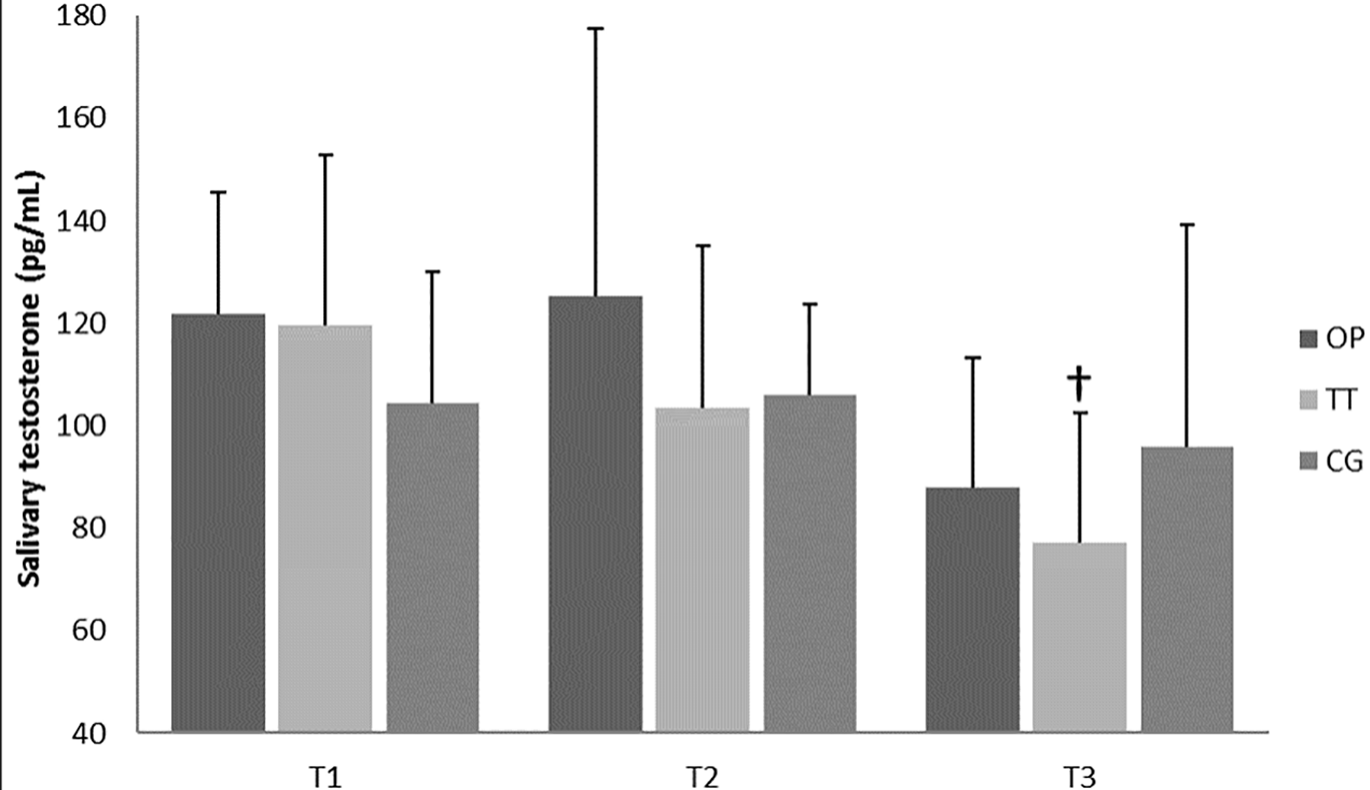
Resting salivary testosterone concentration during main training period. OP = optimum power group; TT = traditional training group; T1 = pre-intervention evaluation; T2 = evaluation after first four weeks’ training; T3 = post-intervention evaluation; † = significant differences from T1 p < .05.

**Fig 4:**
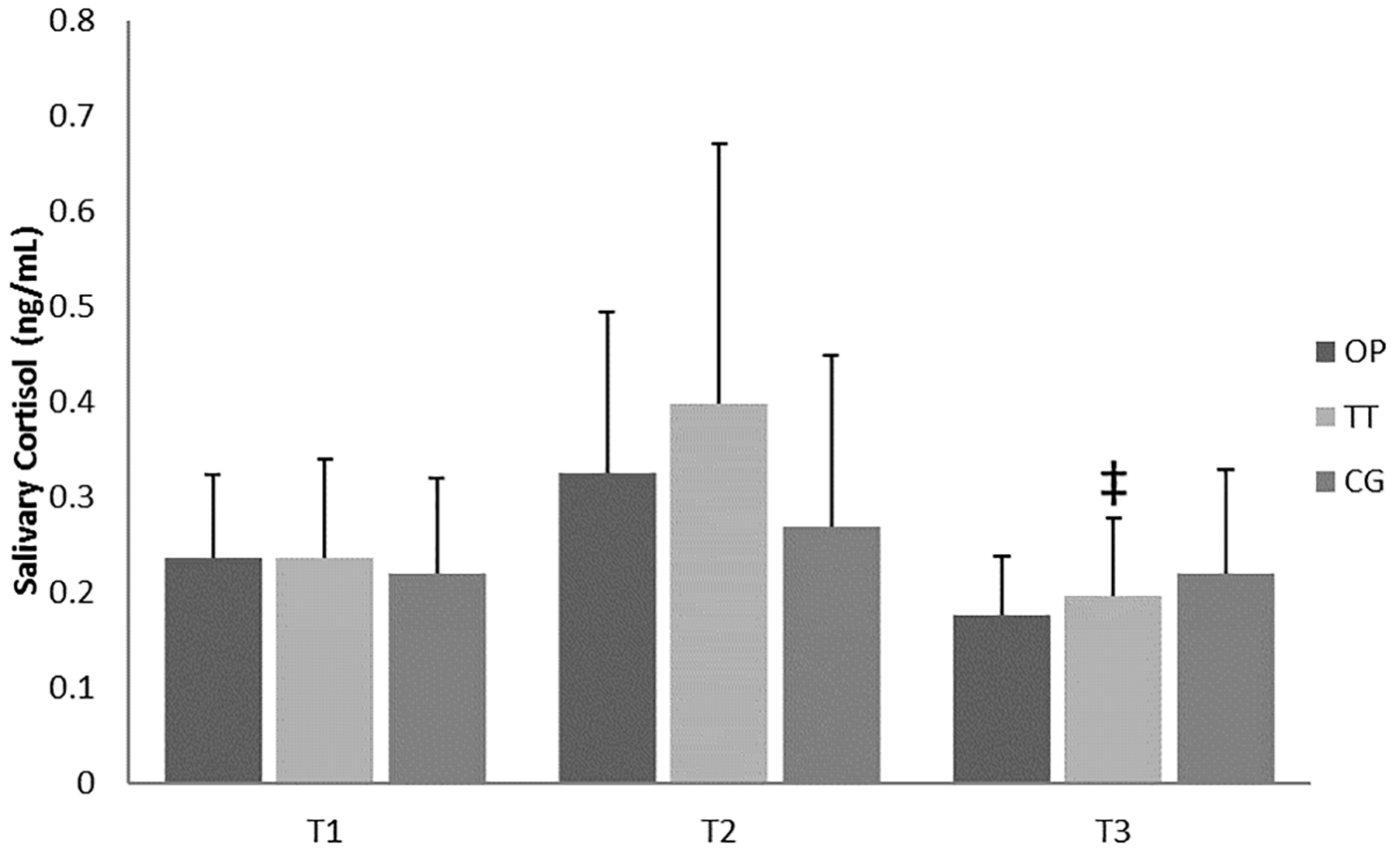
Resting salivary cortisol concentration during main training period. OP = optimum power group; TT = traditional training group; T1 = pre-intervention evaluation; T2 = evaluation after first four weeks' training; T3 = post-intervention evaluation; ‡ = significant differences from T2 p < .05.

The results showed no differences in SC and ST values between groups during the entire intervention period (Figs 3 and 4). However, TT showed significant changes in SC and ST, decreasing from T1 to T3 in the case of ST (*p* = .033, *d* = .43) and from T2 to T3 in the case of SC (*p* = .020, *d* = 1.04).

**Fig 5:**
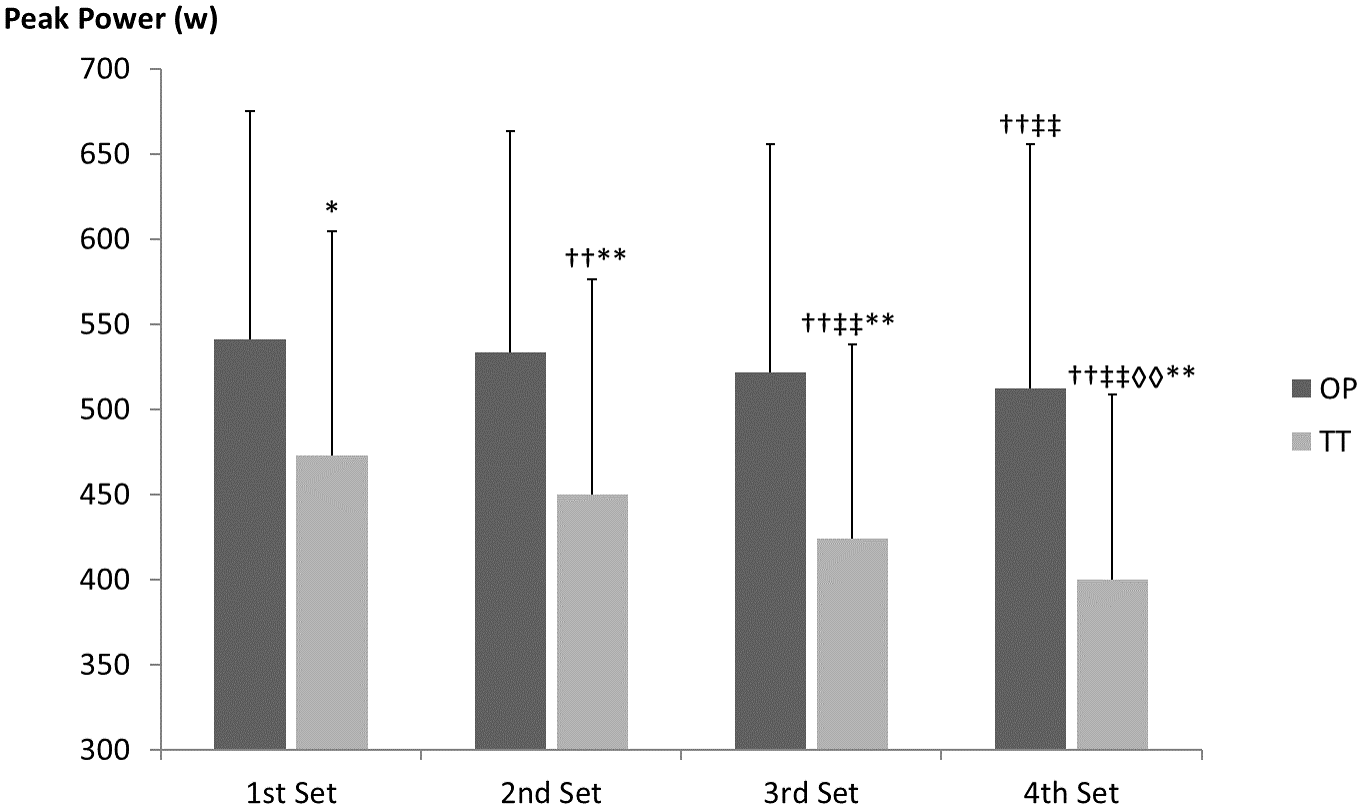
Average in peak power for each set for first two weeks in MESO-2 (sessions with four sets). OP = optimum power group; TT = traditional training group; T1 = pre-intervention evaluation; T2 = evaluation after first four weeks’ training; T3 = post-intervention evaluation; †† = significant differences from 1^st^ set p < .01; ‡‡ = significant differences from 2^nd^ set p < .01; ◊◊ = significant differences from 3^rd^ set p < .01; * = significant differences from OP p < .05; ** = significant differences from OP p < .01.

**Fig 6:**
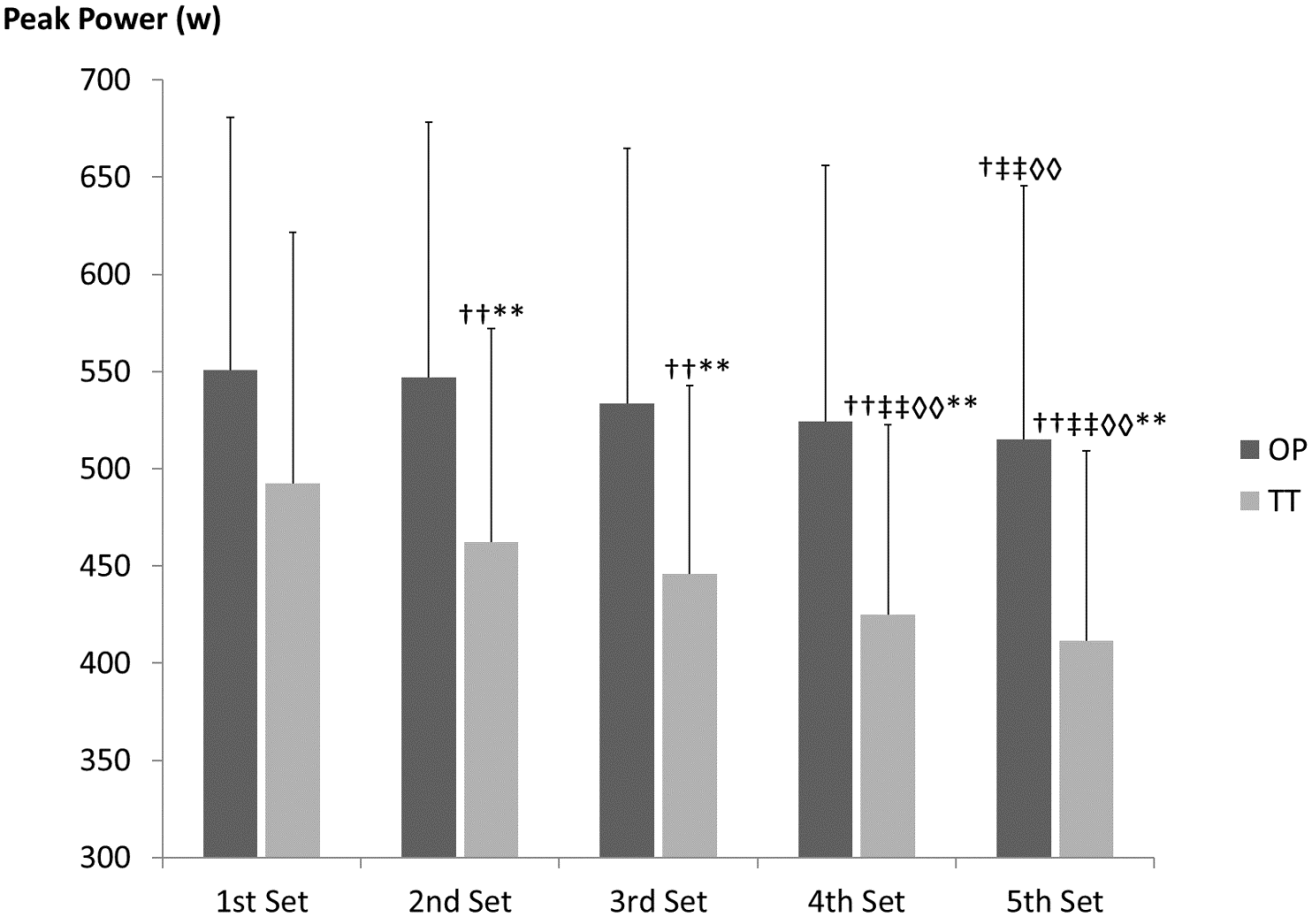
Average in peak power for each set for last two weeks in MESO-2 (sessions with five sets). OP = optimum power group; TT = traditional training group; T1 = pre-intervention evaluation; T2 = evaluation after first four weeks’ training; T3 = post-intervention evaluation; † = significant differences from 1^st^ set p < .05; †† = significant differences from 1^st^ set p < .01; ‡‡ = significant differences from 2^nd^ set p< .01; ◊◊ = significant differences from 3^rd^ set p < .01; ** = significant differences from OP p < .01.

Kinematic data recorded during MESO-2 showed significant decreases in peak power during all sets compared with the 1^st^ set, for TT (2^nd^ set: *p* < .01, *d* = .18; 3^rd^ set: *p* < .01, *d* = .40; 4^th^ set: *p* < .01, *d* = .61), while OP showed a significant decrease only in the last set (4^th^ vs 1^st^ set: *p* = .003, *d* = .21) (see Figs 5 and 6). In addition, data can also be viewed in S1 Table.

TT showed higher values in RPE for both training periods (4.2 and 5.2 for MESO-1 and MESO-2, respectively) compared with OP (2.7 and 3.2 for MESO-1 and MESO-2, respectively), with a significance of p < .01.

## Discussion

The main finding of the current study was that both power training methods are valid for improving power output. Nevertheless, lower RPE and greater control of power loss during sessions were achieved for OP. Moreover, a tendency for quicker improvement in kinematic variables in OP was also found (i.e. from T1 to T2).

Strength and power measures showed that bench press performance was improved for both OP and TT groups after the eight-week training period, with increases in 1RM of 10.6% and 14.5%, respectively. The power output data must be interpreted with caution because the power assessment was from 30 to 50% of 1RM. This range includes the relative load used in the OP training, while the load used in the TT was higher. Although comparisons are difficult because of the use of different methodologies, the present results are similar to those of previous research reporting strength increases in training groups using an ‘optimal load’ approach [3, 39, 40]. Harris et al. [39] evaluated two different seven-week strength training programmes (i.e. loads that maximise power output vs. 80% of 1RM), and found significant improvements in 1RM of 15% and 10.5%, respectively, with no significant differences between groups. More recently, Loturco et al. [40] reported significant improvements in 1RM and power output (i.e. 60% 1RM for back squat and 45% 1RM for jump squat) when using the optimal back-squat load and jump-squat exercises, after a nine-week training period. These changes in 1RM and power were also accompanied by no changes in lean mass, girth measurements of the chest or either relaxed or tensed arm. Thus, it could be hypothesised that improvements in several neural factors that have been shown to influence power output performance, such as motor unit recruitment, firing frequency, motor unit synchronisation and inter-muscular coordination [5], took place.

The present study also showed improvements in power output with 30%, 40% and 50% of 1RM for the OP group, after four weeks of training (i.e. from T1 to T2). To the best of our knowledge, no previous studies have analysed fewer than six weeks of training using a similar training methodology (i.e. optimal load). In this regard, Izquierdo et al. [20], analysing the effects of strength training leading to failure versus not to failure, found no significant changes in bench press power with 60% of 1RM until the eleventh training week (power was evaluated at the 6^th^, 11^th^ and 16^th^ weeks). Therefore, based on the present results we suggest that individualised training loads (i.e. the OP training group) lead to more time-efficient improvements, with an average power output increase of 12% after the first four training weeks and 18% after eight weeks. We can speculate that these faster improvements are due to a reduction in metabolic demands and fatigue [21] caused by the training characteristics, and, therefore, allow higher neural adaptations [41]. In addition to performance measures, hormonal changes showed a tendency of decreasing ST and increasing SC values after four weeks of training for TT, while the OP group tended to show lower levels of SC accompanied by stable or higher levels of ST (i.e. 63% of participants in OP increased their salivary testosterone concentrations from the baseline, while 80% of participants reduced their concentrations in the TT group). These results need to be interpreted with caution, given the controversy in the existing literature. Elevated concentrations of testosterone [42] and cortisol [43] have been reported during strength training, whereas several other studies have shown reductions [42, 44] or no change [45, 46]. Thus, the use of ST and SC remains questionable.

The results of the present study show a progressive performance (i.e. peak power) decrease from the 1^st^ set in the TT group and the highest RPE for the whole period. Similar decreases in power output or velocity have been reported previously [20, 21, 47] associated with muscle glycogen and phosphocreatine (PCr) reductions, particularly in Type II fibres [47], and an increase in RPE [48]. Gorostiaga et al. [21], analysing muscle metabolism during consecutive five-repetition sets with 10RM, found power output decreases (~20%) similar to those in the present study, together with significant changes in PCr, creatine and lactate. In addition, the authors found correlations between peak power output decreases and metabolic parameters (e.g. ATP and lactate). On this basis, Sanchez-Medina and Gonzalez-Badillo [18] concluded that velocity loss (which is directly related to power loss) is a valid tool for quantifying neuromuscular fatigue during strength training. In comparison, the OP in this study suggests that neuromuscular fatigue appeared in the last set of each training session, with a peak power decrease of ~5%, while in TT the 5% power loss occurred in the second set.

The main limitation of the present study is the lack of neural measurements (i.e. surface EMG) that could provide information about neural fatigue in the different training methodologies. Furthermore, given the controversy regarding the differential effects of power training with loads that maximise power output in subjects trained or untrained and more or less strong [49, 50], it is necessary to know the effects of these training methodologies on them. Possibly important differences observed between methods might appear statistically non-significant as a result of the small sample size of the present study. It should be noted that only OP showed increments of power and RM above the MD values, indicating that they could be a real and important change. Nevertheless, further studies are needed to support this hypothesis.

## Conclusions

Individualised power training based on maintaining maximal power output (loads that maximise power output and repetitions mobilised only at maximum power) produces lower intra-session power decreases than do traditionally recommended non-failure sets (50% of the maximum number of repetitions), with less neuromuscular fatigue and similar improvements in power performance, after eight weeks of power training. Therefore, in line with Picerno et al. [13] we recommend the routine use of the encoder for trainers and fitness coaches, with the aim of individualising load training not only in terms of the load used in power training but also the number of repetitions to perform in each set. This will lead to a more efficient training programme by reducing the volume needed (i.e. time or sessions). Thus, training with a load that maximises power output and repetitions mobilised only at maximum power is especially recommended for developing maximum muscular power in short time periods (i.e. around four weeks). This will be very useful in many sports with condensed competitive calendars, where preparatory periods are time limited (i.e. tennis and soccer). On the other hand, this method seems to incur less neuromuscular and metabolic fatigue (i.e. less intra-session power loss and RPE), which can be better for non-trained individuals, because they would be able to bear a lower training load for similar improvements in power.

## Supporting information

S1 File Written informed consent. (DOCX)

S1 Table Raw results data. (XLSX)
