# Supplementary material for: The effects of training with loads that maximise power output and individualised repetitions vs. traditional power training
Source: PLoS One. 2017 Oct 20;12(10):e0186601. doi: 10.1371/journal.pone.0186601 (PMC5650159; doi:10.1371/journal.pone.0186601)
Supplement: S1 File — (DOCX) [file pone.0186601.s001.docx]

**CONSENTIMIENTO INFORMADO PARA EL ESTUDIO “EFECTOS DEL ENTRENAMIENTO DE POTENCIA CON CARGAS Y REPETICIONES OPTIMAS”**

D. .......................................................................................................................... como paciente (o representante), de ............ años de edad, con domicilio en ................................. ..................................................................... DNI nº ............................................

**DECLARO:**

Que el/la Dr./Dra..........................................................................................., me ha explicado que:

Me dirijo a usted como director de un grupo de investigación de la Universidad Miguel Hernández de Elche con la intención de informarle de que deseamos contar con su colaboración para llevar a cabo un estudio que se engloba dentro del proyecto de investigación titulado *“efectos del entrenamiento de potencia con cargas y repeticiones óptimas”*

La habilidad del sistema neuromuscular para generar fuerza de forma rápida es un factor clave en muchos deportes. El objetivo de este proyecto es determinar si la individualización de la carga y las repeticiones de entrenamiento en función de las capacidades del deportista permite desarrollar programas de entrenamiento más eficientes para la mejora de la potencia.

El estudio se llevará a cabo en las instalaciones de la Universidad Miguel Hernández de Elche, y será desarrollado sin utilizar ninguna técnica invasiva. Debido a la intensidad de las sesiones de test y de entrenamientos, siempre existe un riesgo de lesión (no mayor al de las sesiones de entrenamiento habituales). Por ello, cualquier participante con enfermedad cardíaca (cardiopatías, hipertensión, etc.) no deberían formar parte del estudio. Consecuentemente, todos los participantes deben informar sobre cualquier lesión o enfermedad previa.

Los datos obtenidos en este estudio serán usados por el grupo de investigación, quien se compromete a respetar el anonimato y la integridad de los participantes, cumpliendo la ley 15/1999, sobre la protección de datos personales. Los resultados del estudio contribuirán a mejorar los programas de entrenamiento destinados a la mejora de la potencia. Por lo tanto, esperamos contar con su colaboración.

Para cualquier duda o aclaración, puede consultarnos en el número de teléfono:

966 65 88 75, o en la dirección de correo electrónico mmoya@umh.es.

Le saluda atentamente:

Dr. D. Manuel Moya Ramón

Investigador Principal del Proyecto

Observaciones: ...............................................................................................................................

.........................................................................................................................................................

Por ello, manifiesto que estoy satisfecho con la información recibida y en tales condiciones estoy de acuerdo y **CONSIENTO PERMITIR EL USO DE MIS DATOS CLÍNICOS Y DEMOGRÁFICOS PARA INVESTIGACIÓN.**

En .................. de ................................... de 201...

Firma del participante Firma del representante Firma del médico

DNI:

Fdo.: ............................... Fdo.:............................... Fdo.:..................................

(Nombre y dos apellidos) (Nombre y dos apellidos) (Nombre y dos apellidos)

**REVOCACIÓN DEL CONSENTIMIENTO PARA EL ESTUDIO DE INVESTIGACIÓN "EFECTOS DEL ENTRENAMIENTO DE POTENCIA CON CARGAS Y REPETICIONES OPTIMAS"**

D./Dª .................................................................................................................. como paciente (o representante del paciente D.................................................................................................), de ............ años de edad, con domicilio en .................................................................................... ....................................................................................................... DNI. nº ................................. Revoco el consentimiento prestado en fecha...................................... , que doy con esta fecha por finalizado, sin tener que dar explicaciones y sin que esto repercuta en mis cuidados médicos.

En ............. de ................................... de 201...

Firma del paciente Firma de un testigo Firma del médico

DNI:

Fdo.: .......................... Fdo.:............................... Fdo.:....................................

(Nombre y dos apellidos) (Nombre y dos apellidos) (Nombre y dos apellidos)

**INFORMED CONSENT FOR THE STUDY "THE EFFECTS OF TRAINING WITH OPTIMAL LOAD AND REPETITIONS VS. NON-FAILURE POWER TRAINING RECOMMENDATIONS"**

D. .......................................................................................................................... as participant (or legal representative), of ............ years old, with address in ................................. ..................................................................... and identity document nº .........................................

**DECLARE:**

That the Dr............................................................................................, has explained to me that:

I write to you as a headmaster of a research group of the Universidad Miguel Hernández de Elche with the aim of informing about our interest on your participation to carry out a study comprised in a project entitled *“The effects of training with optimal load and repetitions vs. Non-failure power training recommendations”.*

It has been suggested that strength training effects are variable (i.e. neural or structural) depending on the total repetitions performed and velocity loss in each training set. The aim of this study is to compare the effects of different training programmes.

The study will be carried out in the Universidad Miguel Hernández facilities, and will be developed without invasive techniques. Due to the intensity of the testing and training sessions there is always an injury risk (not greater than in the normal training sessions). Anyway, participants cardiovascular diseases (heart disease, hypertensive...) should not took part in this study. Thus, participants should inform about any previous injury/disease.

Data obtained in this study will be used by the research group, who will respect the anonymity and integrity of the participants, accomplishing the law 15/1999, about the Protection of Personal Data.

The result of the study may contribute to improve the programmes of power training. Therefore we hope to count with your collaboration.

If you have any question or doubt, you can contact us by phone (966 65 88 75) or e-mail (mmoya@umh.es).

Sincerely:

Dr. D. Manuel Moya Ramón

Project Headmaster

Note: ...............................................................................................................................

.........................................................................................................................................................

I am satisfied with the information and I agree with **CONSENT THE USE OF MY DEMOGRAPHIC AND CLINIC DATA FOR THIS STUDY.**

In (city) .....................(month)................................ of 201...

Participants signature Legal representative signature Researcher signature

DNI:

Fdo.: ............................... Fdo.:............................... Fdo.:..................................

(Name and surname) (Name and surname) (Name and surname)

**REVOCATION OF THE CONSENT FOR THE STUDY "THE EFFECTS OF TRAINING WITH OPTIMAL LOAD AND REPETITIONS VS. NON-FAILURE POWER TRAINING RECOMMENDATIONS"**

D./Dª .................................................................................................................. as participant (or legal representative of D................................................................................................),of............ years old, with address in.................................................................................... ....................................................................................................... and identification number .................................

Revoke the informed consent given in date...................................... , giving finished this one, without needing to give any explanation.

(city)............. (date) ................................... de 201...

Participants signature Legal representative signature Researcher signature

DNI:

Fdo.: ............................... Fdo.:............................... Fdo.:..................................

(Name and surname) (Name and surname) (Name and surname)
